# Service, science, and fortitude: Our thanks and salute to Dr. Anthony S. Fauci, October 2020

**DOI:** 10.1017/ice.2020.1313

**Published:** 2020-11-05

**Authors:** Judith A. Guzman-Cottrill, David K. Henderson, Hilary Babcock, Sarah Haessler, Mary K. Hayden, A. Rekha Murthy, Clare Rock, Trevor Van Schooneveld, David J. Weber, Sharon B. Wright, Corey A. Forde, Latania K. Logan, Anurag N. Malani

The Board of Trustees of the Society for Healthcare Epidemiology of America express our deep gratitude to Dr Anthony S. Fauci for his tireless and unparalleled leadership of our nation during the coronavirus disease 2019 (COVID-19) pandemic.

As our country moves into the autumn months of 2020, many have described the current COVID-19 pandemic as “unprecedented times.” As we enter what appears to be a third major national surge in infections, hospitalizations, and deaths from COVID-19, this assessment continues to hold true. At the time of this writing, COVID-19 has claimed >220,000 American lives,^1^ resulted in record unemployment,^2^ and it has led millions of students at every educational level into a digital school year.^3^ Projections for the coming winter months suggest that COVID-19 will lead to additional surge-capacity pressures on America’s healthcare system. As infectious disease physicians, we are physically and mentally exhausted. As healthcare epidemiologists, we see no end to our pandemic response. Our healthcare institutions continue to face challenges with personal protective equipment supply chains, limited bed capacity, occupational health risk, pandemic fatigue, financial solvency, and constant modifications in our guidance as new science emerges.

These issues are real, and they are indeed challenging. However, perhaps the most unprecedented problem currently facing our country is not severe acute respiratory coronavirus virus 2 (SARS-CoV-2) or COVID-19 but, rather, a lack of a concerted, coordinated, and strategic public health pandemic response from our nation’s leaders. Much of the leadership responsibility has been abrogated and, instead, delegated to individual states; we now have 50 different, and often inconsistent, approaches. Perhaps even more concerning, the nation’s leaders have not consistently relied on scientific facts to guide planning and response. As with prior epidemics such as human immunodeficiency virus (HIV) and Ebola virus disease, from the beginning of the COVID-19 pandemic, the nation has relied on Dr. Anthony S. Fauci for accurate, science-based recommendations and advice. Dr. Fauci’s nonpartisan commentary is consistently guided by science and, as a result, he is often placed in the unenviable position of having to speak truths that are unwelcome to those in power, which he has done unflinchingly throughout his career and continues to do during this pandemic.

As the SHEA Board of Trustees, we are national leaders in infection prevention and healthcare epidemiology, and the work of the National Institute of Allergy and Infectious Diseases (NIAID) directly affects our own work. Our society would like to publicly thank Dr. Fauci for his calm, consistent reliance on science and for his national leadership during this pandemic. Dr. Fauci is a trusted public health expert who has decades of service. He is a brilliant scientist and is an unwavering leader during our COVID-19 pandemic response.

Dr. Fauci has served as Director of the NIAID since 1984. He has advised 6 US presidents, in both parties, on numerous epidemics, emerging pathogens, and other domestic and international health issues. His contributions to the treatment and prevention of HIV/AIDS, including the President’s Emergency Plan for AIDS Relief (PEPFAR) has saved millions of lives across the world. His expertise and deep dedication to the fields of infectious disease, public health, and epidemiology are unparalleled.

Dr. Fauci remains one of the most trusted infectious diseases physicians and one of the most respected clinical immunologists in the world. From the beginning of the pandemic, he has provided a consistent voice of reason. As the science of SARS-CoV-2 viral transmission dynamics unfolded, Dr. Fauci’s messaging changed accordingly. He continues to share updates with the general public and provides guidance that is evidence-based and pragmatic.

This year has proven to be one of the most tumultuous of our lives and certainly our careers. Despite the current substantial and formidable obstacles to successful public health leadership, Dr. Fauci continues to be a steadfast scientific leader, unwavering in the face of highly visible science denialism. And for that, Dr. Anthony Fauci, we all thank you.
